# The role of family history of preterm delivery in the individual risk of spontaneous preterm delivery: a case–control study

**DOI:** 10.1007/s00404-023-07144-z

**Published:** 2023-07-19

**Authors:** Mor Huri, Noemi Strambi, Marta Finazzi, Giulia Manciucca, Giovanna Catalano, Viola Seravalli, Mariarosaria Di Tommaso

**Affiliations:** Obstetrics and Gynecology Unit, Division of Obstetrics and Gynecology, Department of Health Sciences, University of Florence, Florence, Italy

**Keywords:** Spontaneous preterm delivery, Preterm birth, Family history, Heritability

## Abstract

**Purpose:**

To investigate the role of family history of preterm delivery (PTD) in the individual risk of spontaneous preterm delivery.

**Methods:**

A retrospective case–control study was conducted on 354 patients who delivered between 2018 and 2020. 177 women who delivered preterm were matched with 177 controls who had full-term delivery. A questionnaire was administered to investigate the family history of PTD of both the patient and her partner. Cases and controls were matched for the anamnestic risk factors for PTD.

**Results:**

Seventeen of 173 women (9.8%) in the PTD group reported being born preterm, compared to five of 169 women (2.9%) in the control group (*p* = 0.01), with an odds ratio (OR) of 3.57 (95% confidence interval, CI 1.29–9.92). Women who delivered preterm also reported more frequently having a sibling who was born preterm (12.4% vs. 4.2%, *p* = 0.01), with an OR of 3.18 (95% CI 1.31–7.7). No association was found between the partner’s family history of premature delivery and the patient’s risk of preterm delivery in the present pregnancy.

**Conclusions:**

Pregnant patients who were born prematurely or who have siblings born preterm have an increased risk of preterm delivery in their own pregnancies. Assessment of female personal and family history of PTD should be used to identify women at risk of having a PTD in the present pregnancy.

## What does this study add to the clinical work

**Table:** 

Pregnant patients who were born prematurely or have siblings born preterm have an increased risk of PTD in their own pregnancies.	

## Introduction

Preterm births are those that occur at less than 37 weeks gestational age and are the leading cause of perinatal morbidity and mortality in the developed world [1]. Spontaneous preterm delivery (PTD) results from a complex interaction of genetic, environmental, social, and behavioral factors, although the exact etiology remains unknown [2]. The most important known risk factor is a personal history of preterm delivery, which may be influenced by genetic and environmental determinants [3, 4]. The ability to predict which pregnancies will result in spontaneous PTD is, however, limited [5].

The familial predisposition to PTD has not been well established in literature. Family history of PTD as a risk factor for PTD has been investigated, and although there is some evidence of an intergenerational effect [3, 5–11], this is still controversial and subject of debate. Several data support a genetic predisposition to spontaneous PTD and suggest an intergenerational transmission of PTD risk [3, 5–11]. Other authors, however, have not found similar familial aggregation of PTD [12–15]. Some studies suggest that family history may be considered a risk factor for PTD, but only in association with other anamnestic factors, such as maternal ethnicity and socioeconomic level [16, 17].

A better understanding of the intergenerational transmission of PTD risk can offer opportunities for risk stratification in clinical practice and the creation of prevention strategies [16]. The main purpose of this case–control study was to investigate the role of family history of PTD in the individual risk of spontaneous preterm delivery.

## Methods

This is a mono-center, retrospective, case–control study. The study population was selected by analyzing electronic charts of women hospitalized in the Obstetrics Department of Careggi University Hospital in Florence, Italy, between January 2018 and December 2020. Cases were defined as women who delivered preterm. Controls were defined as women who had a full-term delivery in the same calendar-year, and who did not have a prior history of preterm delivery. To ensure comparability between the two groups, we matched cases and controls for ethnicity, marital status and patient’s education level.

Preterm delivery was defined as delivery before 37 completed weeks of gestation. Exclusion criteria were: multiple pregnancies, iatrogenic preterm deliveries (for indications such as preeclampsia, fetal growth restriction, and other maternal or fetal indications), uncertain gestational age (lack of first trimester ultrasound) and assisted reproductive technology pregnancies with oocyte or sperm donation.

All the patients to be included were contacted by telephone and asked if they agreed to participate in the study. If they gave their informed consent, they were administered a telephone interview using a standard questionnaire to investigate the family history of PTD of both the patient and her partner. In particular, we investigated whether the patient or her partner were born before 37 weeks themselves, as well as if other family members (first- and second-degree relatives) were born prematurely.

The questionnaire included only closed-ended questions (Yes, No, or unknown). The answer "unknown" was selected when the patient did not have full knowledge of her family history of PTD, or her answer was uncertain, and such answer was excluded.

All patients’ answers to the questionnaire were collected in a database along with patients’ and pregnancy characteristics. We obtained information on the following potential confounders from maternal electronic chart records: maternal age, body mass index (BMI), ethnicity, maternal marital status, and maternal education level. Data regarding parity, fetal gender, fetal birth weight and gestational age at delivery were also collected.

Continuous variables were expressed by mean (standard deviation, SD) or medians (interquartile ranges, IQR), depending on the distribution of data; categorical data were expressed by absolute and relative frequencies. Differences between groups were evaluated using the chi square test for categorical data. We calculated the odds ratios (OR) using a two-by-two frequency table. A *p*-value of less than 0.05 was considered statistically significant. Statistical analysis was conducted using statistical software package JMP.

This study was performed according to the principles of the Declaration of Helsinki and was approved by the ethics committee of Careggi University Hospital (reference number 21170_oss). All the data were fully anonymized before being analyzed in a secure database.

## Results

We selected 177 patients who delivered preterm following spontaneous onset of uterine contractions or spontaneous preterm premature rupture of the amniotic membranes. These were matched with 177 controls who delivered at term. Maternal characteristics are illustrated in Table 1. Most patients (88%) were of Caucasian ethnicity.

**Table 1:** Maternal and pregnancy characteristics

	Preterm delivery group (N 177)	Term delivery group (N 177)
Age (years)	34 (31, 37.5)	32.4 ± 5.17
BMI (kg/m^2^)	21.7 (19.9, 24.8)	20.9 (19.5, 23)
Nulliparous	56/177 (31.63%)	79/177 (44.63%)
Ethnicity: White	156 (88.1%)	156 (88.1%)
Black	2 (1.3%)	2 (1.3%)
South-Asian	10 (5.6%)	10 (5.6%)
Hispanic	9 (5.1%)	9 (5.1%)
Marital status: married	107 (60.5%)	107 (60.5%)
Maiden	56 (31.6%)	56 (31.6%)
Divorced/separated	3 (1.7%)	3 (1.7%)
Unknown	11 (6.2%)	11 (6.2%)
Educational level: university degree	79 (44.6%)	79 (44.6%)
High school diploma	63 (35.6%)	63 (35.6%)
Middle school/lower	25 (14.1%)	25 (14.1%)
Unknown	10 (5.6%)	10 (5.6%)
Fetal gender: female	77 (43.5%)	90 (50.8%)
Male	100 (56.5%)	87 (49.2%)
Gestational age at delivery (weeks)	35.1 (30, 36.1)	40 (39.2, 40.5)
Neonatal weight (g)	2365 (1585, 2690)	3361.86 ± 365.48

Data are expressed as mean ± SD or median (1°, 3° IQR) based on their distribution

Table 2 reports the comparison of family history of PTD of the patient and her partner between the groups of cases and controls. Seventeen of 173 women (9.8%) in the preterm delivery group reported being born before term, compared to five of 169 women (2.9%) in the control group (OR 3.57, CI 95% 1.29–9.92, *p*-value 0.01). Women who had delivered preterm reported more frequently having a sibling who was born preterm than controls (12.4% vs. 4.2%, OR 3.18, CI 95% 1.31–7.70, *p*-value 0.01). No statistically significant association was found between patients’ preterm delivery and premature birth of other first or second-degree relatives. Patients whose partner was born prematurely did not have an increased risk of delivering their child prematurely. Similarly, no association was found between the partner’s family history of premature births among siblings or other first or second-degree family members and patients’ risk of preterm delivery in the present pregnancy (Table 2).

**Table 2:** Results

	Preterm delivery group	Term delivery group	Odds ratio (CI 95%)	*p-*value
Patient’s family history				
Patient born preterm	17/173 (9.82%)	5/169 (2.96%)	3.57 (1.29–9.92)	**0.01**
Sibling born prematurely	21/170 (12.35%)	7/165 (4.24%)	3.18 (1.31–7.7)	**0.01**
Other first/second degree relatives born prematurely	24/146 (16.44%)	15/134 (11.19%)	1.56 (0.78–3.12)	0.21
Partner’s family history				
Partner born preterm	11/169 (6.51%)	14/160 (8.75%)	0.72 (0.32–1.65)	0.44
Sibling born prematurely	6/141 (4.26%)	6/122 (4.91%)	0.86 (0.27–2.73)	0.80
Other first/second degree relatives born prematurely	9/141 (6.38%)	8/122 (6.56%)	0.97 (0.36–2.6)	0.95

Bold values are statistically significant (*p*-value < 0.05)

## Discussion

In this study, we aimed to assess if family history of PTD is a risk factor for preterm delivery. Our results showed that women who were born themselves before 37 weeks, or who had siblings born prematurely, were more likely to deliver preterm. This study adds to the body of evidence regarding the intergenerational component of preterm delivery.

Previous studies have shown that mothers born preterm are more likely to have preterm babies. A systemic review and metanalysis demonstrated that maternal preterm birth was associated with their children’s preterm birth [9]. A more recent Canadian retrospective cohort study confirmed such association [10]. Furthermore, a US study demonstrated an inverse relation between maternal gestational age at birth and the individual risk of delivering preterm [3].

Other authors, however, failed to demonstrate such an association. A Danish population-based study found that, after adjusting for maternal characteristics, preterm birth of the mother was not significantly associated with preterm birth of her children [14]. Other two large population-based Scandinavian cohort studies found no significant intergenerational effect of PTD [12, 13]. Similarly, a population study conducted in the United States found no intergenerational transmission of preterm delivery among non-Hispanic Whites and African Americans [15].

Transmission of the PTD risk from a mother to her offspring does not imply necessarily a genetic etiology, as socioeconomic factors such as parental education, maternal age, and marital status can also be similar between family members [12, 14]. A US population-based study found an intergenerational effect of PTD among non-Hispanic Black mothers but not among non-Hispanic White mothers [17]. Another study conducted in the United States suggested that race and generational socioeconomic contexts modify intergenerational transmission of PTD risk [16]. In our study, however, patients with and without preterm delivery were matched for ethnicity, marital status and patient’s education level, thus eliminating the influence that such confounders have on the association studied.

Our results also showed that women who have siblings born prematurely are at increased risk of delivering preterm. These results are in accordance with the findings of previous studies. A Swedish population-based study found that mothers with an older sister who had given birth to a preterm infant had an 80% higher risk of delivering preterm themselves [8]. Furthermore, a Canadian study demonstrated that mothers born at term with a sister born preterm had a similarly elevated risk of delivering a preterm infant as their preterm sisters [7].

Other lines of evidence support a female family history as a risk factor for PTD. A large Scottish retrospective study found the risk of spontaneous PTD was increased in daughters whose mothers had a history of a similar type of delivery in any pregnancy [6]. A recent US retrospective cohort study showed that spontaneous PTD in the current pregnancy was significantly associated with a maternal family history of preterm delivery among female relatives within 3 generations [5]. Similarly, an Israeli study identified female family history of PTD as an independent risk factor for preterm delivery [18].

Our results did not identify a significant role of the paternal factor in the risk of PTD. The results are consistent with other studies that suggested the lack of genetic contribution from the father to the PTD risk. A Danish population-study demonstrated that a change in the female partner was associated with a reduction in the recurrence risk of having a child born prematurely [19]. A change in the male partner, on the other hand, did not reduce the risk of delivering preterm for mothers who previously had a preterm child, which points toward the major importance of maternal factors [20]. Two large Norwegian population-based studies found that mothers born preterm, but not fathers, have an increased relative risk of having a child born preterm [21, 22]. This suggests that fetal genotype may be of relatively little importance in understanding the genetic patterns of preterm delivery, since fetal genes that increase the risk of preterm delivery would be transmitted through both parents [21].

The biological mechanisms for the observed intergenerational associations are not fully known, but could be explained by genotype, epigenetic and environmental mechanisms [16]. Maternal genotype has been suggested to affect the length of gestation in a genome-wide association study, and polymorphism in genes such as those encoding proinflammatory cytokine were associated with spontaneous preterm delivery [23–27]. Epigenetics, which reflects the interaction of environmental stressors with underlying genetic susceptibility, is another potential mechanism for these intergenerational PTD associations [16, 25]. Mitochondrial fetal genes, transmitted solely through the maternal line, are another possible explanation [11, 21].

The strengths of the present study include the accurate selection of eligible patients, with exclusion of cases of iatrogenic preterm delivery, and the fact that we investigated the influence of both the maternal and paternal family history on the risk of PTD.

A limitation of our study was its relatively small sample size. Despite that, we were able to find statistically significant associations.

We acknowledge that the recurrence of specific pregnancy outcomes in successive generations might also be due to mothers and daughters experiencing the same environmental risk factors. Preterm delivery is known to be associated with socioeconomic factors such as parental education, maternal age when giving birth, and marital status [12]. Therefore, confounding bias is the most important bias to consider. We tried to minimize this potential bias by matching cases and controls for maternal characteristics such as ethnicity, marital status and maternal education level. Our study may also be limited by a recall bias, as the participants were asked to answer questions about their parents’ and relatives’ history.

In conclusion, women who were born prematurely, or have siblings born preterm, have an increased risk of delivering preterm in their own pregnancies. A patient’s personal and family history of PTD should be collected at the time of pre-conception or first antenatal visit in order to identify women at risk of having a PTD and to plan for an appropriate counselling and follow-up.

Further studies should aim to identify possible effects of environmental or genetic factors on PTD recurrence across generations. This will lead to a more specific characterization of risk profiles associated with PTD and ultimately to improved prevention strategies.

## Data Availability

The data that support the findings of this study are available from the corresponding author, upon reasonable request.
